# Paternal Mitochondrial DNA Leakage in Natural Populations of Large-Scale Loach, *Paramisgurnus dabryanus*

**DOI:** 10.3390/biology13080604

**Published:** 2024-08-10

**Authors:** Zixin Qi, Jiaoxu Shi, Yue Yu, Guangmei Yin, Xiaoyun Zhou, Yongyao Yu

**Affiliations:** 1 College of Animal Science and Technology, Henan University of Animal Husbandry and Economy, Zhengzhou 450046, China; 2 College of Fisheries, Huazhong Agricultural University, Wuhan 430070, China; 3 Agronomy and Life Science Department, Zhaotong University, Zhaotong 657000, China; 4 Hubei Hongshan Laboratory, Wuhan 430070, China

**Keywords:** *P. dabryanus*, heteroplasmy, mitochondrial DNA, paternal leakage

## Abstract

**Simple Summary:**

Mitochondrial DNA is generally thought to strictly follow maternal inheritance, meaning that vertebrates have just one type of mitochondrial DNA haplotype. However, a very interesting phenomenon occurs in a fish species, *Paramisgurnus dabryanus* (*P. dabryanus*), regarding the presence of two distinct mitochondria in one individual. Phylogenetic analysis suggested that interspecific hybridization may occur between *P. dabryanus* and *Misgurnus anguillicaudatus*, leading to the transfer of mitochondrial DNA from the father to their offspring. The investigation of natural populations across regions demonstrated that this species commonly possesses two types of mitochondria. Furthermore, qPCR analysis suggested that type I plays a major role. The results of this study help us better understand how animal mitochondrial DNA can differ due to paternal contribution, giving us useful ideas about how mitochondrial genomes evolve and are passed down.

**Abstract:**

Animal mitochondrial DNA is usually considered to comply with strict maternal inheritance, and only one mitochondrial DNA haplotype exists in an individual. However, mitochondrial heteroplasmy, the occurrence of more than one mitochondrial haplotype, has recently been reported in some animals, such as mice, mussels, and birds. This study conducted extensive field surveys to obtain representative samples to investigate the existence of paternal inheritance of mitochondrial DNA (mtDNA) in natural fish populations. Evidence of paternal mitochondrial DNA leakage of *P. dabryanus* was discovered using high-throughput sequencing and bioinformatics methods. Two distinct mitochondrial haplotypes (16,569 bp for haplotype I and 16,646 bp for haplotype II) were observed, differing by 18.83% in nucleotide sequence. Phylogenetic analysis suggests divergence between these haplotypes and potential interspecific hybridization with *M. anguillicaudatus*, leading to paternal leakage. In natural populations of *P. dabryanus* along the Yangtze River, both haplotypes are present, with Type I being dominant (75% copy number). Expression analysis shows that Type I has higher expression levels of *ND3* and *ND6* genes compared to Type II, suggesting Type I’s primary role. This discovery of a species with two mitochondrial types provides a model for studying paternal leakage heterogeneity and insights into mitochondrial genome evolution and inheritance.

## 1. Introduction

Mitochondria are important organelles in eukaryotes that provide energy for cellular processes [1]. The mitochondrial DNA (mtDNA) is the extranuclear genetic material independent of the replication control of nuclear DNA. According to the prevailing consensus, mitochondria originated from α-proteobacteria. Most of the genes in the ancestral bacterial genome during evolution were lost or transferred to the nucleus, leaving only the compact mtDNA molecule [2]. Thus, in sexual reproduction, mitochondria and mtDNA are normally inherited exclusively from the mother (maternally inherited) [3], enabling genealogical researchers to trace maternal lineage far back in time. Combining with its small size (generally 15–20 kb), high copy number, and higher mutation rate (compared to nuclear genes), mtDNA has thus been transformed as a crucial molecular tool widely used in early species identification (i.e., DNA barcoding) and phylogenetic reconstruction studies [4,5].

Mitochondrial heteroplasmy, i.e., the occurrence of more than one mitochondrial haplotype, has been demonstrated in a broad range of species, both vertebrates and invertebrates [6,7,8]. Mitochondrial heteroplasmy can be generated by somatic mutations within an individual or heteroplasmy of the oocytes [7,9], including length or site variation, i.e., the presence of different mtDNA lengths or different nucleotide orders, respectively, in the same cell. While site heteroplasmy is rare, length heteroplasmy, the occurrence of different size variants due to variable numbers of tandem repeats in the noncoding control region [10,11], is frequent in natural populations. Such length variation is thought to have arisen through slipped strand mispairing during replication [12].

In addition, heteroplasmy can also be generated by paternal leakage of mtDNA, where paternal mitochondrial DNA is not always eliminated during egg fertilization [13]. Male mitochondrial DNA inheritance has been discovered in Plymouth Rock chickens [14]. There is also evidence suggesting rare instances of male mitochondrial inheritance in certain mammalian species. Specifically, there are documented occurrences in mice [15], although the mitochondria inherited from the male were ultimately rejected. This phenomenon has also been observed in sheep [16] and, in rare cases, humans [17,18]. Doubly uniparental inheritance of mtDNA is observed in bivalve mollusks. In those species, females have only one type of mtDNA (F), whereas males have F-type mtDNA in their somatic cells but M-type mtDNA (which can be as much as 30% divergent) in germline cells [19]. Paternally inherited mitochondria have additionally been reported in some insects, such as fruit flies [20], honeybees [21], and periodical cicadas [22]. Moreover, paternal leakage has so far been well-documented within hybrid zones of intersecting species or populations in sea turtles [23], birds [24], and insects [25]. Currently, paternal inheritance of mtDNA has not been reported in natural fish populations except in artificially hybridized species under laboratory conditions [26,27].

Large-scale loach, *P. dabryanus* (Cobitidae, Cypriniformes), is a small freshwater teleost that inhabits the muddy bottom of creeks, ponds, wetlands, and paddy fields. Through high-throughput sequencing, phylogenetic analysis, and field investigation of natural populations, we showed evidence of paternal mtDNA leakage in natural populations of large-scale loach, *P. dabryanus,* by sequencing the entire mitochondrial genomes. We found two distinct mitochondria coexist in one individual. By reconstructing the phylogenetic relationship of the Cobitoidea fishes, we found that these two mitochondrial haplotypes belong to separate, divergent species groups. The one haplotype (Type I) considered as the type itself of *P. dabryanus* clusters with *Misgurnus nikolskyi* and *Misgurnus mohoity*, while the other (type II) clusters closely with *M. anguillicaudatus*. Interestingly, this phenomenon of dual mitochondrial inheritance is prevalent in the natural population of this species with higher copies of type I. These results indicate the occurrence of interspecific hybridization and paternal mtDNA leakage between *M. anguillicaudatus* and *P. dabryanus* in nature and the retention of dual mtDNA in the offspring.

## 2. Materials and Methods

### 2.1. Experimental Animals

Healthy *P. dabryanus* were collected from various populations in the upper, middle, and lower reaches of the Yangtze River basin, China, including Erhai (ER,14 individuals, 21.0–33.3 g), Dongting Lake (DT, 15 individuals, 22.5–35.5 g), Honghu Lake (HH, 15 individuals, 18.0–31.0 g), Liangzi Lake (LZ, 17 individuals, 20.2–32.1 g), and Taihu Lake (TH, 20 individuals, 19.6–28.4 g). Loach species was identified according to Chen and Zhu [28].

### 2.2. RNA Extraction and cDNA Synthesis

The total RNA from various tissues of healthy *P. dabryanus* was extracted using the TRIZol reagent according to the manufacturer’s instructions. The concentrations of the total RNA were carried out by NanoDropND-1000 spectrophotometer (Thermo Scientific, Wilmington, DE, USA), and the integrity of tissue RNA was determined by agarose gel. After MS-222 anesthetized the fish, blood was drawn from the tail vein, and the tissues, including the brain, heart, fin, kidney, spleen, gut, gill, and skin, were collected. Genomic DNA was extracted using the standard phenol-chloroform method.

### 2.3. Polymerase Chain Reaction (PCR) Amplification and Sequencing

The mitogenomes of *P. dabryanus* were amplified using the long PCR method. Two fish-versatile primers (S-LA-16S-L+S-LA-16S-H) [29] were used to amplify the entire mitochondrial genome in a single reaction. The reaction was performed with 25 µL reaction volume containing 10 × LA PCR buffer II (Mg^2+^), 1.25 mM of dNTPs, 0.5 mM of each primer, 1.25 µL LA Taq polymerase (Takara, Beijing, China), and approximately 100 ng template DNA. The thermal cycle profile was pre-denaturation at 94 °C for 1 min, followed by 30 cycles of 98 °C for 10 s, 68 °C for 16 min, and finally with 72 °C for 10 min. To further validate the sequence obtained above, two sets of other fish-versatile primers (S-LA-16S-L+H15149-CYB and L12321-Leu+S-LA-16S-H) were used to amplify the mitogenome of the same fish in two fragments of ~12 and ~7 kb. Unfortunately, a high level of nucleotide divergences was observed in the overlapping region of the two segments, making it impossible to reach the full target genome sequence. The sequence amplified using S-LA-16S-L+H15149-CYB was the same as a corresponding region with S-LA-16S-L+S-LA-16S-H (16S to Leu). However, the sequence amplified using L12321-Leu+S-LA-16S-H exhibited extensive divergence with the corresponding region (16S to Cytb) of S-LA-16S-L+S-LA-16S-H. Therefore, new primer pairs (P3F+P5R, 3.5 kb; P5F+P7R, 2.5 kb; P7F+P9R, 3 kb; P9F+P13-L, 5.5 kb) were designed to amplify the region of L12321-Leu+S-LA-16S-H, and the PCR conditions were the same as previously described. For distinguishing, this haplotype was designated as Type II, while the former was Type I. To determine whether the observed cases were random events, 30 fish from the same area were selected randomly for further examination.

The long PCR products were sequenced using a primer walking strategy with primer pairsP1-P18. These primers were designed based on sequence alignment of 26 mtDNA sequences of Cobitoidea species, retrieved from GeneBank (Table 1).

The standard PCR reaction was performed with a 10 µL reaction volume containing 7.7 μL of ddH_2_O, 0.1 µL of Taq polymerase (Takara), 1 µL of Taq buffer, 0.2 µL of dNTPs, 0.25 µL of each primer, and 0.5 µL of DNA template. Gradient PCR is performed, followed by agarose gel electrophoresis of the amplification products. Bands are visualized on a gel imaging system to determine the optimal annealing temperature for each primer design, with a clear and bright band indicating the best condition. The thermal cycle profile was pre-denaturation at 94 °C for 2 min, followed by 32 cycles of 94 °C for 30 s, 55–65 °C for 30 s, 72 °C for 40 s, and finally with 72 °C for 5 min. The sequencing was conducted by Sangon Biotech (Shanghai, China) Co., Ltd.

For quantitative PCR (qPCR), amplification was performed with the specific primer pair targeting fin’s mitochondrial DNA *ND3* and *ND6* gene in a 10 µL reaction mix, which contained 5 µL of Hieff qPCR SYBR Green Master Mixv (No Rox) and 0.15 µL of each primer, DNA 2 µL, and PCR-grade water 2.7 µL. The reactions were carried out following the procedure: 95 °C for 30 s, followed by 41 cycles of 95 °C for 1 s, 55 °C for 10 s, and then 72 °C for 6 min. Table 2 lists the primer pairs used in this experiment. Mitochondrial DNA copies were calculated according to the standard curve, which was constructed based on a series of diluted plasmids containing the *ND3* and *ND6* genes and were expressed with copies per µL DNA (copies/µL DNA).

### 2.4. Gene Annotation and Sequence Analysis

The sequence fragments obtained were edited in the Seqmen program (DNAstar, Madison, WI, USA) for contig assembly to obtain the complete mitogenome sequences. DOGMA software was used to annotate protein-coding and ribosomal RNA genes and define their respective gene boundaries [30]. The tRNAs and their secondary structures were identified by tRNAscan-SE 1.21 software [31]. The putative origin of light strand replication (OriL), control region (CR), and conserved motifs were identified via sequence homology. Sequences were aligned using the Clustal W [32]. The numbers of polymorphic sites, nucleotide, and amino acid divergence between the two haplotypes were estimated with DnaSP v5.0 [33].

For the construction of the mitochondrial gene map of *P. dabryanus*, we employed the following processes: genes encoded on the heavy or light strands are shown outside or inside the circular gene map, respectively. The inner ring represents the GC content. Both mtDNA types of *P. dabryanus* possess a uniform gene arrangement and similar gene sizes. The figure was initially generated with pDRAW32 and subsequently modified manually.

### 2.5. Phylogenetic Analyses

To understand the genetic relatedness of these two haplotypes, the complete mitochondrial genome sequences of the two haplotypes in this study, together with previously reported mitogenome sequences of 26 Cobitoidea species as well as *Danio rerio* and *Cyprinus carpio* (outgroup) available from GenBank were used to perform phylogenetic analysis. The trees were constructed using the maximum likelihood (ML) and Bayesian inference (BI) methods, using MEGA version 5.0 and MrBayes 3.1.2 [34], respectively.

The jModelTest program [35] was used to determine the best-fitting models of nucleotide substitution. The Akanke’s Information Criterion (AIC) indicated that the GTR+I+G model is the most appropriate for each dataset. The following settings were applied in the BI analyses: the number of Markov chain Monte Carlo (MCMC) generations = three million, sampling frequency = 1000, and burn-in = 250. The robustness of the resultant ML tree was evaluated using bootstrap probabilities calculated from nonparametric bootstrap analyses with 500 pseudo-replications.

The *COX2* genes of *P. dabryanus* from Erhai (EH), Dongting Lake (DT), Liangzi Lake (LZ), and Honghu Lake (HH) were obtained and sequenced to understand the phylogeny of mtDNA haplotypes. The phylogenetic tree of the *COX2* gene of *P. dabryanus* was constructed using the Neighbor-joining (N-J) method in the MEGA 6.0 software.

### 2.6. Statistical Analysis

An unpaired Student’s t-test (Prism version 6.01; GraphPad) was used for the analysis of differences between mitochondrial DNA haplotype I and haplotype II copy numbers in the same tissue. *p*-values of 0.05 or less were considered statistically significant.

## 3. Results

### 3.1. Mitogenome Organization and Composition

**Figure 1:**
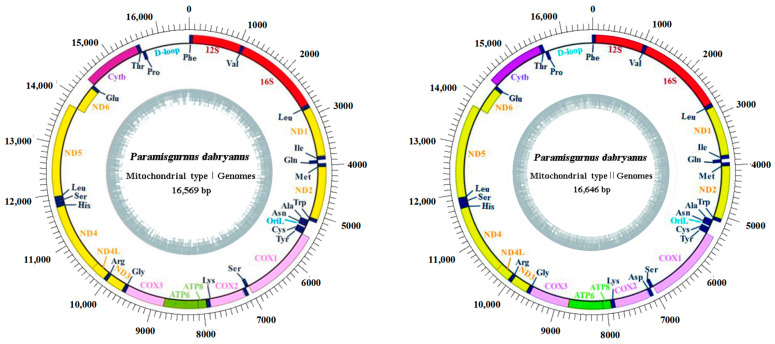
Gene map of *P. dabryanus* mitochondrial genome.

Two distinct mitochondrial haplotypes were sequenced from one *P. dabryanus* fish. The two mtDNAs exhibit typical vertebrate mitochondrial genomes with identical gene content and gene arrangement. They comprise a heavy chain (H-chain) and a light chain (L-chain); both of them share an identical gene composition and encode 37 genes in total, including 13 protein-coding genes, 22 tRNA genes, 2 rRNA (12S rRNA and 16S rRNA), and two major non-coding regions—the control region and the origin of light-strand replication (OriL). Except for the *ND6* gene and eight tRNA genes (tRNA^Gln^, tRNA^Ala^, tRNA^Asn^, tRNA^Cys^, tRNA^Tyr^, tRNA^Ser^, tRNA^Glu^, and tRNA^Pro^), the remaining 28 genes are all located on the heavy chain (Figure 1). However, the lengths of the complete genome were 16,569 bp and 16,646 bp for Type I and Type II, respectively; the Type II genome is 73 nucleotides longer than Type I. The most prominent difference is the presence of a 71-bp insertion consisting of the last 44 bp of *COX2* and 27 bp of intergenic spacer following the *COX2* gene, which was not found in Type I. After examining 30 samples, each product used was amplified using primer N7F+N9L primer as a template and then amplified using primer N8F+N8L primer; all can identify the presence of this segment. 

Furthermore, the arrangement order, gene length, and the use of start and stop codons of all genes are highly similar. Apart from the control region, there are only 12 non-coding intergenic spacers, ranging from 1 to 27 bp in length. The largest intergenic spacer is 27 bp, located between the Type II tRNA^Lys^ and 12S rRNA gene. Additionally, some adjacent genes overlap with the overlap length ranging from 1 to 10 bp. The longest overlap, 10 bp, occurs between the *ATPase8* and *ATPase6* genes. The total length of the 13 protein-coding genes in the mitochondrial genome of *P. dabryanus* ranges from 11,433 to 11,472 bp. Except for the *COX1* gene, which starts with GTG as its start codon, the remaining 12 protein-coding genes initiate with ATG as their start codon. In Type I, the *COX2*, *COX3*, and *Cytb* protein-coding genes terminate with T, while the remaining 10 genes possess the typical complete stop codon TAA. In Type II, apart from the *ND2*, *COX3*, *ND3*, and *Cytb* genes that terminate with T and the *ND4* gene that terminates with TA, the other eight protein-coding genes exhibit the typical complete stop codons TAA or TAG (Table 3).

**Figure 2:**
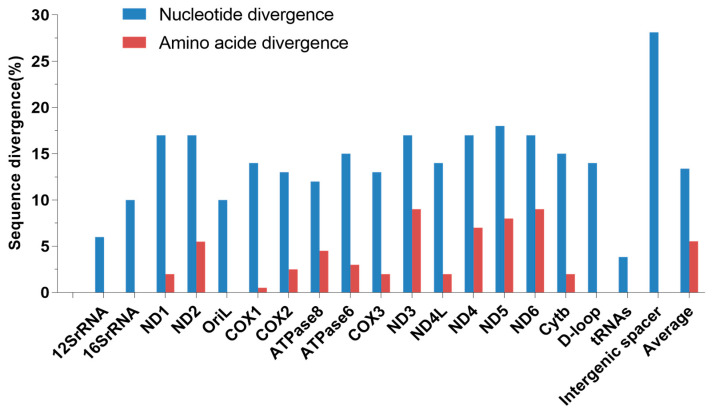
The nucleotide and amino acid divergence between the two mtDNA haplotypes were sequenced from one *P. dabryanus* fish. Note: The blue bars represent the nucleotide divergence; the red bars represent amino acid divergence.

Besides the large insertion leading to obviously longer in Type II, the two haplotype sequences exhibit high sequence divergences in all genes/regions, highest in the intergenic spacer (28.13%) and lowest in the tRNAs (3.85%); the overall nucleotide and amino acid divergence were 13.38% and 5.54%, respectively (Figure 2).

### 3.2. Phylogenetic Relationships

**Figure 3:**
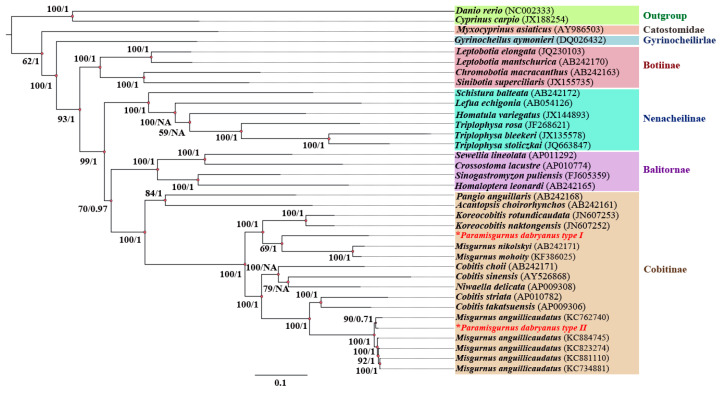
Phylogenetic analyses of the two mtDNA haplotypes sequenced from one *P. dabryanus* fish and the other 28 Cobitoidea species. Note: The phylogenetic analyses were conducted based on the complete mtDNA sequences with maximum likelihood (ML) and Bayesian inference (BI) methods. Numbers on the nodes represent support values inferred from ML (left) bootstrap and BI (right) probability analyses, respectively. * Represents the two mitochondrial haplotypes detected in this study.

The ML and BI phylogenetic trees of complete mtDNA from the present two haplotypes and 28 Cobitoidea species were reconstructed and exhibited similar phylogenetic topologies (Figure 3). As expected, the species from Cobitinae, Balitornate, Nemacheilinae, and Botiinae were monophyletic and formed one clade, respectively, which is consistent with the phylogenetic studies based on the morphological data [36] and molecular biology [37]. However, the two mitochondria haplotypes of *P. dabryanus* fell into two divergent phylogenetic groups. Type I was clustered with *M. nikolskyi* and *M. mohoity* and then with the genus Koreocobitis. Contrarily, Type II was clustered with five-level ploidy *M. anguillicaudatus*, especially for tetraploids, which our laboratory sequenced, and then clustered with the genus Cobitis with high bootstrap supports (Bootstrap value = 100, BP = 1).

### 3.3. Investigation of Mitochondrial Heterogeneity in P. dabryanus from Different Regions

**Figure 4:**
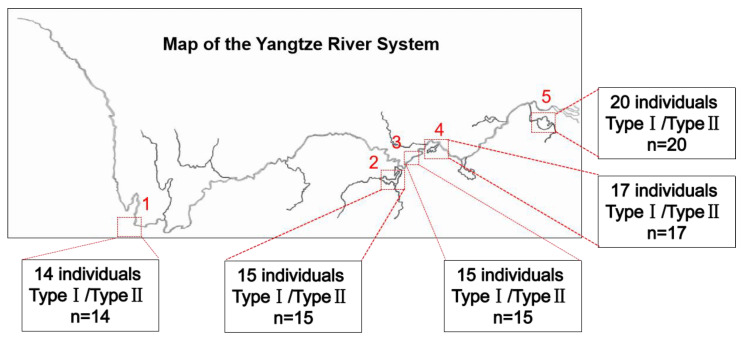
Investigation on mitochondrial heterogeneity of wild populations of *P. dabryanus.* Note: 1 represents Erhai (EH); 2 represents Dongting Lake (DT); 3 represents Honghu Lake (HH); 4 represents Liangzi Lake (LZ); 5 represents Taihu Lake (TH); Type I/Type II signifies the presence of two distinct mitochondrial haplotypes within a single fish.

This study conducted an investigation into the natural populations of *P. dabryanus* in the Yangtze River System, the largest river system in China, to gain insights into the distribution and occurrence frequency of the two types of mitochondria in natural populations. Sampling sites covered various regions in the upper, middle, and lower reaches of the Yangtze River. A total of 81 samples were collected from 5 sampling sites, with the largest number, 20, from Taihu Lake (TH) and the smallest number, 12, from Liangzi Lake (LZ). Regions with significant differences in the *COX2* gene between two haplotypic mitochondria were amplified utilizing previously screened stable primers. The detection results indicated that wild populations located in Erhai (EH) upstream, Liangzi Lake (LZ) midstream, Taihu Lake (TH) downstream, and two lakes connected to the main and tributary streams all contained two haplotypes. In simpler terms, despite significant habitat differences, natural populations in different regions are composed of fish with two haplotypes of mitochondria (Figure 4).

**Figure 5:**
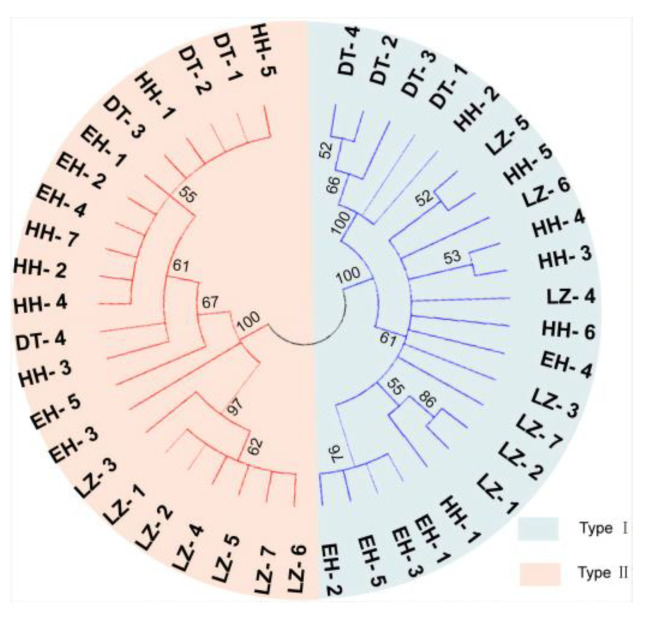
Phylogenetic analyses of the two mtDNA haplotypes sequenced from different regions. Note: The blue part represents Type I, and the red part represents Type II.

Phylogenetic analyses based on the *COX2* gene revealed that natural populations in different regions are composed of fish with two haplotypes of mitochondria (Figure 5).

### 3.4. Tissue Distribution and Expression Analysis of Two Mitochondrial DNA Haplotypes

**Figure 6:**
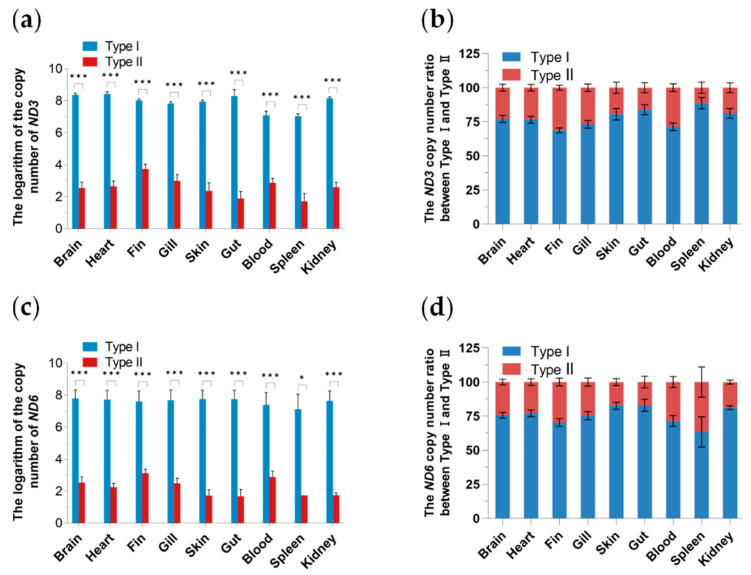
Mitochondrial DNA copy numbers and ratios in tissues. Note: (**a**) The copy number of *ND3* gene (*n* = 10); (**b**) The *ND3* copy numbers ration between Type I and Type II; (**c**) The copy number of *ND6* gene (*n* = 6); (**d**) The *ND6* copy numbers ration between Type I and Type II. * *p* < 0.05, *** *p* < 0.001. Data are representative of three independent experiments (mean ± SEM).

*ND3* and *ND6* proteins are crucial for the assembly and function of oxidative phosphorylation (OXPHO) complex I (C I) of the mitochondrial electron transport chain that produces adenosine triphosphate (ATP), which is essential for life. Variants in *ND3* and *ND6* genes can cause severe functional defects in mitochondria. In this study, the mitochondrial DNA copy number was determined through the *ND3* and *ND6* genes. Overall, regardless of the mitochondrial copy number determined by the mitochondrial *ND3* or *ND6* gene, both Type I and Type II distributions were observed in 9 tissues, with the copy number of Type I being significantly (*p* < 0.001) higher than that of Type II. The contents of Type I and Type II in blood and spleen tissues were lower than those in other tissues, and the mitochondrial DNA copy numbers of both types were the lowest in the spleen. In the same tissue, the proportion of Type I copy number was as high as approximately 75% (Figure 6).

**Figure 7:**
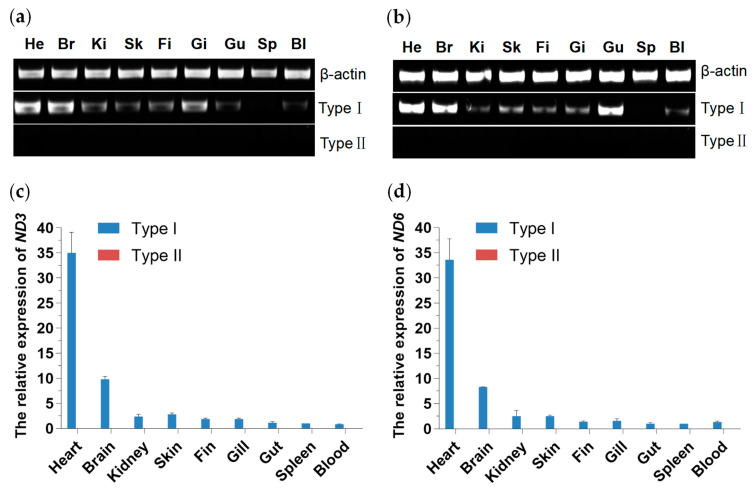
The relative expression of mitochondrial genes *ND3* and *ND6*. Note: (**a**) The gel image of the *ND3* gene; (**b**) The gel image of the *ND6* gene; (**c**) The relative expression of mitochondrial gene *ND3*; (**d**) The relative expression of mitochondrial gene *ND6*. Note: He represents Heart; Br represents Brain; Ki represents Kidney; Sk represents Skin; Fi represents Fin; Gi represents Gill; Gu represents Spleen; Bl represents Blood.

As shown in Figure 6, both mitochondrial DNA haplotypes are distributed in all tested tissues. This study conducted an expression analysis of mitochondrial genes *ND3* and *ND6* to investigate the functions of these two types of mitochondria. As illustrated in Figure 7, the *ND3* and *ND6* genes of Type I mitochondria are expressed in all tested tissues, with higher expression levels in heart and brain tissues compared to other tissues. The expression level in the heart is the highest, approximately more than three times that in the brain. However, no expression of *ND3* and *ND6* genes was detected in Type II haplotype across all tissues.

## 4. Discussion

Mitochondrial DNA is usually considered to comply with strict maternal inheritance, with only one mitochondrial DNA haplotype in vertebrates. Male mitochondrial DNA inheritance and doubly uniparental inheritance of mtDNA are observed in some animals. Here, we found that the phenomenon generally exists in a species of fish, *paramisgurnus dabryanus*, regarding the presence of two distinct complete mtDNA in one individual. We identified the complete mitochondrial genome length of *P. dabryanus* to be 16,569 bp (Type I) and 16,646 bp (Type II), respectively. It revealed that the gene composition and arrangement were consistent with typical vertebrate mitochondrial genomes, with Type I exhibiting a similar length to the two previously reported mitochondrial genomes of *P. dabryanus* [38,39]. However, Type II is reported for the first time, similar to *M. anguillicaudatus*. Only ATG and GTG were identified as start codons among the mitochondrial protein-coding genes of *P. dabryanus*, a phenomenon commonly observed in the mitochondrial DNA of other animals [40,41,42]. Among the 13 protein-coding genes in *P. dabryanus*, six genes terminated with complete stop codons (TAA and TAG), while the remaining genes terminated with T or TA. Incomplete stop codons may form complete stop codons through polyadenylation during mRNA processing [43]. The overall nucleotide and amino acid differences in the two haplotype sequences are beyond the range expected for conspecific mtDNA. Sequence divergence usually averages 1 to 3% within species, even though some can be as high as 10% [44]. For example, the overall mean divergence of four human mitochondrial genomes (Cambridge Reference Sequence [NC_001807], a Swedish [X93334], an African [D381112], and a Japanese [AB055387]) is 0.5%; the mean divergence for the 26 complete genomes of *Drosophila simulans* [45] is 1.6% [46]. Thus, the high levels of divergence at the interspecific level between the two haplotypes indicate that the heteroplasmy observed in these specimens is more likely due to leakage of paternal mitochondrial DNA rather than base substitutions or slipped strand mispairing, which mainly occurred in the noncoding control region.

Previous researchers showed that paternal leakage is often associated with hybrid zones [47], where the reproduction barriers between hybridizing species or populations may not be efficient at preventing paternal mtDNA inheritance [6]. Additionally, fertilization experiments in fruit flies and cattle showed that survival of paternal mitochondria might be higher in interspecific crosses than within-species mating because molecular recognition systems may be relaxed in such crosses [48,49]. *P. dabryanus* and *Misgurnus anguillicaudatus* are found sympatrically in most areas in China with similar morphological characters and nearly overlapping spawning seasons (March to October) [50]. Hybridization between *P. dabryanus* and *M. anguillicaudatus* has been identified at relatively high frequencies in wild populations [50]. Although *P. dabryanus* × *M. anguillicaudatus* hybrids may display reduced fecundity, leakage of mtDNA would be possible if the hybrids between *P. dabryanus* and *M. anguillicaudatus* were fertile because the F1 hybrids might backcross to their parental generation as shown in other loaches [51,52]. Thus, it seems reasonable to assume that the *P. dabryanus* fish employed in the present study resulted from interspecific hybridization between *P. dabryanus* and *M. anguillicaudatus*, and the heteroplasmy observed in these specimens is probably due to leakage of paternal mtDNA resulting from interspecific hybridization.

The Yangtze River is the first largest river in China and originate from the Qinghai-Tibet Plateau. Its mainstream and tributary flow through 11 provincial-level administrative units from west to east. The diverse climatic types, complex topographic features, and unique hydrological and aquatic environments of the Yangtze River have contributed to a rich variety of habitats. Assessing the evolutionary significance of paternal inheritance also necessitates understanding its frequency and consequences in natural populations [53]. Field population surveys have revealed that the wild populations throughout the Yangtze River basin are both of the dual-mitochondrial type. Increasing evidence suggests that paternal inheritance of mtDNA exists in various species, including mice [15], fish [26], ticks [54], and nematodes [6], but this phenomenon is typically observed only in laboratory or controlled settings. Consequently, the frequency of this phenomenon might have been underestimated, especially in natural populations. In contrast, this study investigated the natural populations of *P. dabryanus* in different basins of the Yangtze River system in China. All fish collected from representative upstream, midstream, and downstream regions possessed two mitochondrial types, indicating a 100% frequency of paternal leakage. It is acknowledged that as the sampling scope expands and the sample size increases, the observed frequency may decrease. Nevertheless, as previously mentioned, the frequency of paternal leakage could still be underestimated. This finding suggests that dual-mitochondrial populations occupy a relatively high frequency in wild populations and can be inherited stably.

Many copies of mtDNA are in one cell. In the homoplasmic state, a cell or a tissue has mtDNA of only one genotype, whereas, in a heteroplasmic state, there is not just one genotype [2]. In this study, we investigated the wild populations of *P. dabryanus* in 5 representative regions of the Yangtze River, encompassing a total of 81 fish, all of which contained two distinct mitochondrial types. To delve deeper into the distribution pattern of these two mitochondrial types within fish, we conducted qPCR analysis on wild populations of *P. dabryanus* collected at Liangzi Lake (LZ). The results revealed that, across all tissues sampled, except for the gonads, the copy number of mitochondrial Type I was significantly higher than that of Type II, with the highest ratio of copy numbers between the two types approximating 3:1. Regarding a single mitochondrial type, tissues other than blood and spleen exhibited higher copy numbers. Organs with high energy requirements, such as the heart and brain, have been proven to harbor high mtDNA copy numbers, whereas tissues like the spleen, which rely on an alternative metabolic pathway—glycolysis—tend to possess fewer mtDNA copies [55]. Notably, despite the presence of both mitochondrial DNA types in all fish, the Type II mitochondrial genes remained unexpressed. The regulation of mtDNA expression is highly complex, involving multiple layers of control. Apart from its own genes and regulatory elements, mitochondria rely on various nuclear genome products to fulfill important functions such as replication, transcription, and translation [2]. The big-scale loach (*P. dabryanus*) belongs to the Cypriniformes, Cobitidae, and Paramisgurnus genera and is known for its high survival rate, strong disease resistance, and rapid growth rate [56]. However, whether the emergence and stable inheritance of the two types of mitochondria are related to the strong adaptability of *P. dabryanus* remains to be further explored. Meanwhile, the mechanisms and significance of paternal leakage of animal mitochondrial DNA and the relationship between evolution and function deserve more attention.

## 5. Conclusions

In conclusion, the sequencing and phylogenetic analyses using the complete mtDNA sequences revealed two distinct mitochondrial haplotypes in one *P. dabryanus* fish. The two mtDNA sequences exhibit similar gene content and gene arrangement despite the fact that their primary DNA sequence has diverged by 13.376%. More importantly, phylogenetic analysis of the Cobitoidea indicated interspecific hybridization between *P. dabryanus* and *M. anguillicaudatus* in natural populations, and subsequently, mitochondrial DNA leakage occurred in their offspring. We investigated the natural populations across various regions of the Yangtze River and found that, despite significant habitat differences, these populations in the disparate regions are consistently composed of fish possessing two distinct haplotypes of mitochondria. Through this research, we have obtained detailed information about the complete sequence of the mitochondrial DNA genome of *P. dabryanus* and discovered possible clues for its paternal mitochondrial inheritance, which lays a foundation for further exploring the relationship between the structural heterogeneity and function of the mitochondria. In all, our findings provide a fish model for studying the heterogeneity of paternal leakage mitochondria and also offer valuable insights into the evolution and inheritance of mitochondrial genomes.

## Figures and Tables

**Table 1:** PCR and sequencing primers designed for amplifying the mitochondrial genome of *P. dabryanus*.

Forward	Sequence (5′–3′)	Reverse	Sequence (5′–3′)
**Long PCR Primers**			
**S-LA-16S-H**	TGCACCATTRGGATGTCCTGATCCAACATC	**S-LA-16S-L**	CGATTAAAGTCCTACGTGATCTGAGTTCAG
**L12321-Leu**	GGTCTTAGGAACCAAAAACTCTTGGTGCAA	**H15149-CYB**	GGTGGCKCCTCAGAAGGACATTTGKCCTCA
**P13-L**	CCAGCAATAATACTTCCTCAG		
**Internal Primers**			
**P1F**	CACTGAAGATGCTAAGATGG	**P1R**	TCTCTGCCTGTTGTATGC
**P2F**	TTACACCGAGAAGACATCC	**P2R**	CTCGTCTTGTAGGTGTATGC
**P3F**	CAGTGACCACAAGTTCAAC	**P3R**	GCATATTCAGCCAGGAAGA
**P4F**	TCTCTAGCCTTGCCGTATA	**P4R**	GTTGATAGGATTAGACCTGTTG
**P5F**	ATAGCACAGCAGCATCAC	**P5R**	GCAGTTCCAACCATTCCA
**P6F**	AACTTAGACCAAGAGCCTTC	**P6R**	CACGAGTATCAACATCTATTCC
**P7F**	ACTGCCGTTCTTCTTCTAC	**P7R**	AGCCTAAGTCCTCATAGTCA
**P8F**	ATAACCAYTCTGCCAYTTC	**P8R**	CGATTRATTAGTCAGCCTTG
**P9F**	CGTTCCACTTGAGCACTT	**P9R**	ATGGTCAGAAGAAGCAGAAT
**P10F**	GTCTATTCATTCGTCCATTAGC	**P10R**	GCATTGTAGGAGATTGAGGTT
**P11F**	AAGACCGTGGTTCAACTC	**P11R**	TGTGTTCGCTCGTAAGTG
**P12F**	TGTTCACCTCTGACTACCTA	**P12R**	CATCTGCTCGTCCGTATC
**P13F**	CATCCTGATAYATRCACTCTGAC	**P13R**	CCAGCAAYAATACTTCCTCAG
**P14F**	CTGGCATTCCTTCACATCT	**P14R**	TTCTTCAAGTCACTGGTCTC
**P15F**	CTGCCACCACTAATCCTAA	**P15R**	GCTCACTCTAATGCCTTGT
**P16F**	GCCACTATTCTACATCTACTCT	**P16R**	GCATAAGTCAACACCTACTG
**P17F**	AACAAGGCATTAGAGTGAG	**P17R**	GTATGACAGCCAAGAGGT
**P18F**	GATACCAGTAGAACATCCAT	**P18R**	GCCCTCTTATCCCTAACTA

**Table 2:** Primers for mitochondrial heterogeneity and mitochondrial DNA copy numbers detection.

Forward	Sequence (5′–3′)	Reverse	Sequence (5′–3′)
**Two Types of Mitochondrial Detection Primers**			
**Test1-F**	ACCCGTACTTATACTAAAACC	**Test1-R**	CGATGCCAATAGAACAGC
**Test2-F**	CCGTACTTATACTAAAACCG	**Test2-R**	CAAGAAGATTATTAGGGAGG
**Test3-F**	GCTAGAAATAGCAACTATGG	**Test3-R**	GGGAGGTGAGTAGTAGGG
**Test4-F**	CGTTCCACTTGAGCACTT	**Test4-R**	ATGGTCAGAAGAAGCAGAAT
**Test5-F**	TATCGTCGCCATAGTCTCCA	**Test5-R**	GTTTTAGTATAAGTACGGGTTTT
**Test6-F**	CAACTATGGAAGAAGTTACA	**Test6-R**	CAAGCAGGGTTAATAGGAAT
**Test7-F**	TACTTTACATTATCGTCGCC	**Test7-R**	GTTTTAGTATAAGTACGGGTTTT
**Test8-F**	TACTTTACATTATCGTCGCC	**Test8-R**	CTTCCATAGTTGCTATTTCT
**Quantitative Real-Time PCR Primers**			
**ND3-1F**	CCTTGGATCTGCTCGTTT	**ND3-1R**	CCCTGAGCCCACTCGTAT
**ND3-2F**	GATCTGCCCGATTACCAT	**ND3-2R**	CATTCATAAACCAAGCCTAA
**ND6-1F**	TGGTTGCTGTGGCTTCTA	**ND6-1R**	GCCAGAGCTGCCGAATAG
**ND6-2F**	CTGGCCGCTGAACCATTT	**ND6-2R**	CTCCTCGTAACATACTAAATTCC

The test represents primers for mitochondrial heterogeneity detection; *ND3*-1 represents primers for Type I mitochondrial DNA copy numbers detection; *ND3*-2 represents primers for Type II mitochondrial DNA copy numbers detection. The *ND6* gene primers are the same.

**Table 3:** Characteristics of *P. dabryanus* Type I and Type II mitochondrial genomes.

Name of Gene	Location		Size (bp)	Start Codon	Stop Codon	Intergenic Nucleotides	Stand	Nucleotide Divergence
	I	II	I/II	I/II	I/II	I/II	I/II	I to II
tRNA^Phe^	1–69	1–69	69			0	H	0.05
12S rRNA	70–1023	70–1022	954/953			0	H	0.06
tRNA^Val^	1024–1095	1023–1094	72			0	H	0.03
16S rRNA	1096–2773	1095–2773	1678/1679			0	H	0.1
tRNA^Leu(UUR)^	2774–2848	2774–2848	75			1	H	0.01
ND1	2850–3824	2850–3824	975	ATG	TAA	7/6	H	0.17
tRNA^Ile^	3830–3901	3831–3902	72			−2	H	0.04
tRNA^Gln^	3900–3970	3901–3971	71			1	L	0
tRNA^Met^	3972–4040	3973–4041	69			0	H	0.03
ND2	4041–5087	4042–5086	1047/1045	ATG	TAA/T--	−2/0	H	0.17
tRNA^Trp^	5086–5155	5087–5156	70			2/1	H	0.08
tRNA^Ala^	5158–5226	5158–5226	69			1	L	0.04
tRNA^Asn^	5228–5300	5228–5300	73			0	L	0.01
OriL	5301–5330	5301–5330	30			0	L	0.1
tRNA^Cys^	5331–5396	5331–5396	66			0	L	0.08
tRNA^Tyr^	5397–5465	5397–5465	69			1	L	0.03
COX1	5467–7017	5467–7017	1551	GTG	TAA	1	H	0.14
tRNA^Ser(UCN)^	7019–7089	7019–7089	71			0/2	L	0.06
tRNA^Asp^	7090–7161	7092–7163	72			3/13	H	0.07
COX2	7175–7865	7177–7911	691/735	ATG	T--/TAA	0/27	H	0.13
tRNA^Lys^	7866–7941	7939–8014	76			1	H	0.03
ATPase8	7943–8110	8016–8183	168	ATG	TAA	0/−10	H	0.12
ATPase6	8101–8784	8174–8857	684	ATG	TAA	−1	H	0.15
COX3	8784–9567	8857–9640	784	ATG	T--	0	H	0.13
tRNA^Gly^	9568–9640	9641–9713	73			0	H	0.03
ND3	9641–9991	9714–10,062	351/349	ATG	TAA/T--	−1/0	H	0.17
tRNA^Arg^	9991–10,060	10,063–10,132	70			0	H	0.06
ND4L	10,061–10,357	10,133–10,429	297	ATG	TAA	−7	H	0.14
ND4	10,351–11,733	10,423–11,804	1383/1382	ATG	TAG/TA-	−1/0	H	0.17
tRNA^His^	11,733–11,801	11,805–11,873	69			0	H	0
tRNA^Ser(AGY)^	11,802–11,869	11,874–11,941	68			0/1	H	0.04
tRNA^Leu(CUN)^	11,871–11,943	11,943–12,015	73			0	H	0.01
ND5	11,944–13,782	12,016–13,854	1839	ATG	TAA/TAG	−4	H	0.18
ND6	13,779–14,300	13,851–14,372	522	ATG	TAA	0	L	0.17
tRNA^Glu^	14,301–14,369	14,373–14,441	69			7/6	L	0
Cytb	14,377–15,517	14,448–15,588	1141	ATG	T--	0	H	0.15
tRNA^Thr^	15,518–15,589	15,589–15,660	72			−2	H	0.06
tRNA^Pro^	15,588–15,657	15,659–15,728	70			0	L	0.03
D-loop	15,658–16,569	15,729–16,646	912/918				H	0.14

T-- and TA- represent incomplete stop codons; positive numbers represent nucleotides that separate adjacent genes; negative numbers represent overlapping nucleotides; and 0 indicates that adjacent genes are exactly contiguous.

## Data Availability

The original contributions presented in the study are included in the article, further inquiries can be directed to the corresponding authors.
